# Supplementary material for: Evaluation of Discrimination Performance in Case for Multiple Non-Discriminated Samples: Classification of Honeys by Fluorescent Fingerprinting
Source: Sensors (Basel). 2020 Sep 18;20(18):5351. doi: 10.3390/s20185351 (PMC7570986; doi:10.3390/s20185351)
Supplement: Supplementary file 1 [file sensors-20-05351-s001.pdf]

Supplementary Information to paper

Evaluation of discrimination performance in case for multiple non-discriminated samples: classification of honeys by fluorescent fingerprinting

Elizaveta A. Rukosueva, Valeria A. Belikova, Ivan N. Krylov, Vladislav S. Orekhov, Evgenii V. Skorobogatov, Andrei V. Garmash and Mikhail K. Beklemishev

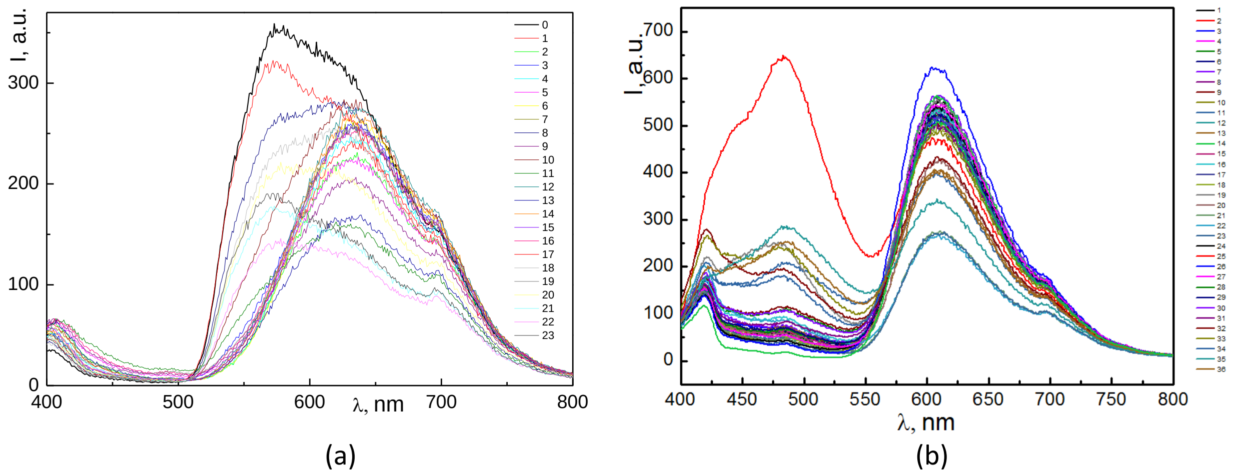

Figure S1. Fluorescence spectra of honey samples with added fluorophores: (**a**) intercalated thiazole orange (TO, ratio DNA-2 : TO = 1 : 105, λ_{ex} = 320 nm). With large amounts of the dye (TO:DNA ratios greater than 1:53), the intrinsic fluorescence of honey is quenched, and only a TO emission peak is observed in the spectrum. The largest number of discriminated groups was achieved with the TO:DNA-2 ratio of 1:53; (**b**) $Ru(bpy)_3^{2+}$ (50 μL of 1×10^{-5} mol/L solution added to each well). The outlaying red spectrum belongs to a deeply colored sample.

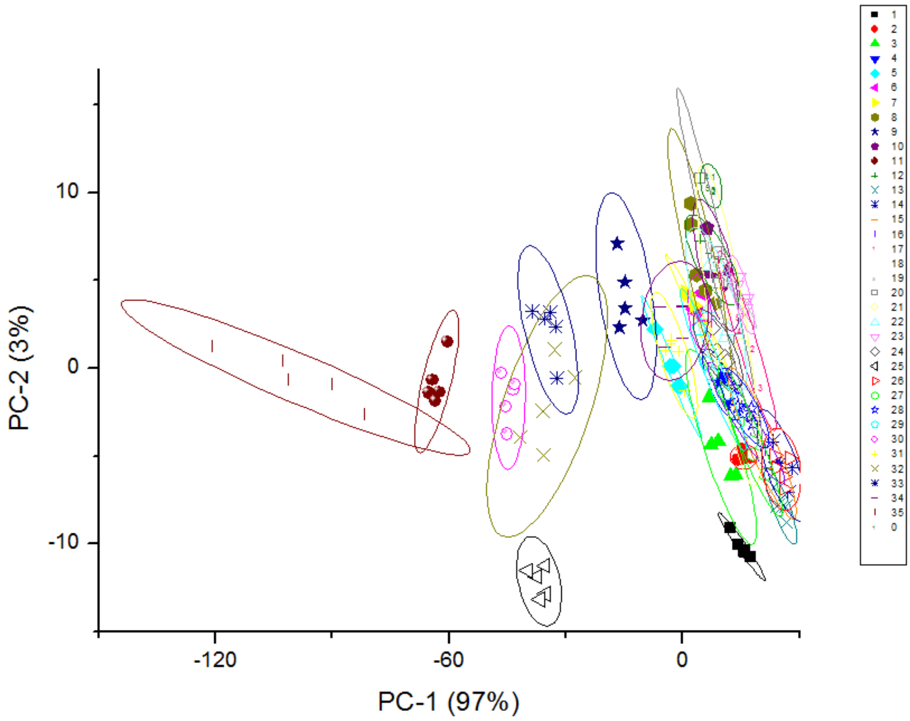

(a)

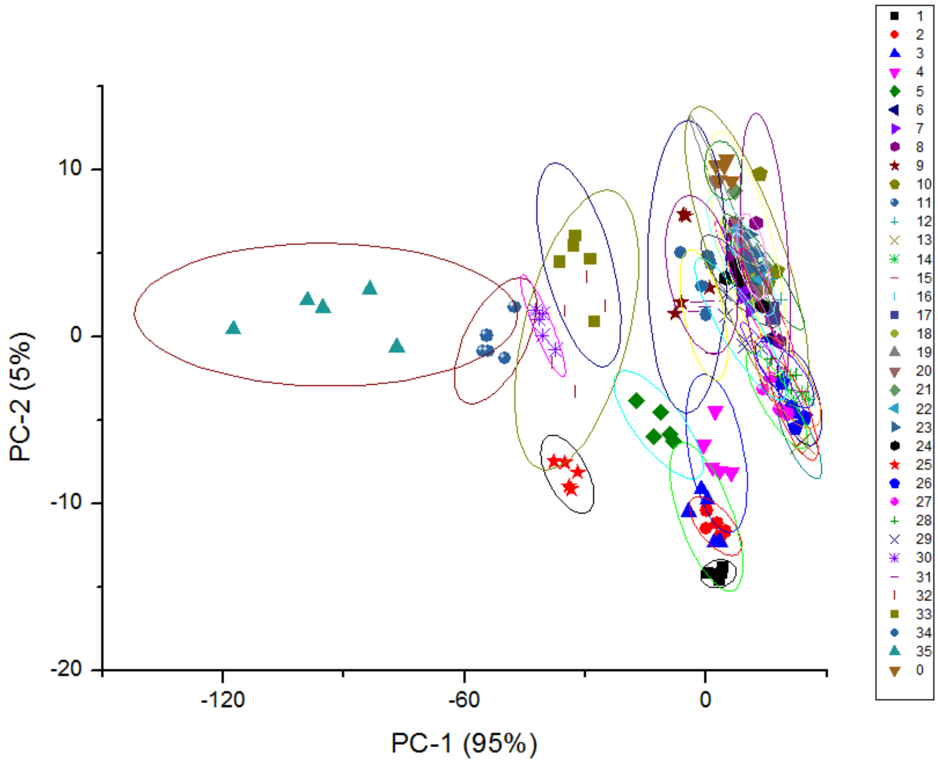

(b)

Figure S2. Scores plots of the visible region reflection images of honey samples in the 96-well plates: (**a**) without added Ru(bpy)$_3^{2+}$, (**b**) in the presence of Ru(bpy)$_3^{2+}$. Conditions are the same as used in fluorescence studies.

Table S1. The number of discriminated groups of points in the scores plots of 23 honey samples with added thiazole orange (TO) intercalated into DNA-2 (TO–DNA-2 fluorophore). The groups were considered separated if their confidence ellipses did not intersect .

**Composition of the Blend (Ratio TO:DNA)**	**Number of Discriminated Groups of Points in the Scores Plot**		
	**by Full Spectrum (400-800 nm)**	**by One Peak**	
		**Shortwave**	**Longwave**
Honey	7	8	–
Honey + DNA	11	10	–
Honey + DNA + TO (1:6)	10	8	5
Honey + DNA + TO (1:21)	8	5	5
Honey + DNA + TO (1:53)	9	–	12
Honey + DNA + TO (1:105)	4	–	6
Honey + DNA + TO (1:210)	4	–	5

Standard formulas for the calculation of confidence ellipses (based on the instruction to Origin software)

Assuming the pair of variables (X, Y) conforms to a bivariate normal distribution, we can examine the correlation between the two variables using a confidence ellipse. The confidence ellipse is centered at $(\bar{x},\ \bar{y})$ (for a given dataset (x_i, y_i), $i\ =\ 1, 2, \dots n$, where x is the independent variable and y is the dependent variable), and the major semiaxis a and semiaxis b equal:

$$\bar{x} = \frac{1}{n}\sum_{i=1}^{n} x_i,\ \bar{y} = \frac{1}{n}\sum_{i=1}^{n} y_i,$$

$$a = c\sqrt{\frac{\sigma_x^2+\sigma_y^2+\sqrt{(\sigma_x^2-\sigma_y^2)+4r^2\sigma_x^2\sigma_y^2}}{2}},\ b = c\sqrt{\frac{\sigma_x^2+\sigma_y^2-\sqrt{(\sigma_x^2-\sigma_y^2)+4r^2\sigma_x^2\sigma_y^2}}{2}},$$

where $\sigma_i^2$is the variance equal.

For a given confidence level of (1-α) the confidence ellipse is defined as:

$$c = \sqrt{\frac{2(n+1)(n-1)}{n(n-2)}\left(\alpha^{\frac{2}{2-n}} - 1\right)}.$$

The inclination angle of the ellipse is defined as:

$$\beta = \frac{1}{2} arctan\frac{2r\sqrt{\sigma_x^2\sigma_y^2}}{\sigma_x^2-\sigma_y^2}.$$

Matlab function for relative position (RP) calculation (composed by the authors)

```
function[RP] = relative_position (sc1,sc2)
% sc1 - double [K1,2], where K1 is count of points in first class
% sc2 - double [K2,2], where K2 is count of points in second class
c1 = mean(sc1);% C1 - the center of the first group
c2 = mean(sc2);% C2 - the center of the second group
sc1 = get_border(sc1);% any border - ellipse or convex polygon
sc2 = get_border(sc2);
    % find A1,A2
    for i = 1:size(sc1,1)
        proj1(i) = (c1-c2)*(sc1(i,:)-c2)'/(sqrt((c1-c2)*(c1-c2)'));
    end
    for i = 1:size(sc2,1)
        proj2(i) = (c2-c1)*(sc2(i,:)-c1)'/(sqrt((c1-c2)*(c1-c2)'));
    end
    [~,a1] = min(proj1);
    a1 = sc1(a1,:);
    [~,a2] = min(proj2);
    a2 = sc2(a2,:);
    % find D1,D2
    di1 = find_d(a1,c1,c2);
```

```
        di2 = find_d(a2,c1,c2);
        % find RP
        c1c2 = sqrt((c1-c2)*(c1-c2)');
        c2d2 = sqrt((c2-di2)*(c2-di2)');
        c1d1 = sqrt((c1-di1)*(c1-di1)');
        RP = (c1c2-c2d2)/c1d1;
end

function[res] = find_d(a,ci1,ci2)
        eps = 10^(-5);
        n1 = ci2(1) - ci1(1);
        n2 = ci2(2) - ci1(2);
        if abs(n2) < eps && abs(n1) < eps
                res = a;
        elseif abs(n2) < eps
                res = [a(1),ci1(2)];
        elseif abs(n1) < eps
                res = [ci1(1),a(2)];
        elseif abs(n2-1) < eps
                de1 = (a(1)*n1^2+a(2)*n1-ci1(2)*n1+ci1(1))/(n1^2+1);
                de2 = a(2)-n1*(de1-a(1))/n2;
                res = [de1,de2];
        else
                e = -1/n1-n1;
                g=n2-1/n2;
                f=ci1(2)/n2-ci1(1)/n1-n1*a(1)-n2*a(2);
                de1 = (a(1)*n1+a(2)*n2+n2*f/g)/(n1+n2*e/g);
                de2 = a(2)-n1*(de1-a(1))/n2;
                res = [de1,de2];
        end
end


function[nodes] = get_border (X)%convex polygon
        X = unique(X,'rows');
        if size(X,1) == 2
                nodes = X;
        else
                [~,i] = min(X(:,2));
                nodes(1,:) = X(i,:);
                X(i,:) = [];
                for i = 1:size(X,1)
```

```
            angle(i) = acot((X(i,2)-nodes(1,2))/(X(i,1)-nodes(1,1)));%acot == arc cotangent
        end
        [angle,i] = sort(angle,'descend');
        X = X(i,:);
        X(end+1,:) = nodes(1,:);
        nodes = [X(end-1,:);nodes;X(1,:)];
        i = 2;
        while i <= length(angle)+1
            if isLeft(nodes(end-2,:),nodes(end-1,:),nodes(end,:)) > 0
                nodes(end+1,:) = X(i,:);
                i = i + 1;
            else
                nodes(end-1,:) = [];
            end
        end
    end
end

function[res] = isLeft(x0,x1,x2)
    res = ((x1(1)-x0(1))*(x2(2)-x0(2)) - (x2(1)-x0(1))*(x1(2)-x0(2)));
end

function[res] = dist(x)
    res = sqrt(x(1)^2+x(2)^2);
end
```
